# Nutritional Comparison of Sacha Inchi (Plukenetia volubilis) Residue with Edible Seeds and Nuts in Taiwan: A Chromatographic and Spectroscopic Study

**DOI:** 10.1155/2022/9825551

**Published:** 2022-09-19

**Authors:** Sahri Yanti, Dinesh Chandra Agrawal, Dinar Suksmayu Saputri, Hung-Yu Lin, Wei-Jyun Chien

**Affiliations:** ^1^Department of Applied Chemistry, Chaoyang University of Technology, 168, Jifeng E. Rd., Wufeng District, Taichung City 413310, Taiwan; ^2^Department of Agricultural Product Technology, Sumbawa University of Technology, Olat Maras Rd. Sumbawa District, West Nusa Tenggara 84371, Indonesia; ^3^Department of Environmental Engineering and Management, Chaoyang University of Technology 168, Jifeng E. Rd., Wufeng District, Taichung City 413310, Taiwan

## Abstract

Sacha inchi is a source of quality commercial oil in Taiwan. Oil extraction results in sacha inchi residue have not been utilized and not much investigated. Different edible seeds and nuts have different levels of nutrients. This study aims (a) to determine the oil, moisture, ash, protein, carbohydrate, type of fatty acid, resveratrol, and type of sugar in edible seeds and nuts, including sacha inchi residue, and (b) to determine the model to predict the five macronutrients using NIR spectroscopy. The samples used were candlenut, peanut, sesame, sunflower, sacha inchi residue, and black bean. Determination was conducted using NIR spectroscopy, NMR spectroscopy, LC-MS/MS, and HPLC-ELSD. NIR spectroscopy prediction results show that candlenut is rich in oil, and sacha inchi residue is rich in minerals, protein, and moisture. The correct prediction model for oil and moisture is principal component regression, while partial least squares are for ash, protein, and carbohydrates. NMR spectroscopy results showed that all samples were rich in polyunsaturated fatty acids. Sacha inchi residue is rich in omega 3. LC-MS/MS results showed that all samples contained resveratrol, and its highest level was found in sesame. HPLC-ELSD results showed eight types of sugars in the samples. High sucrose was found in sacha inchi residue, sunflower, sesame, and candlenut. The results are expected to provide information on nutrient levels in seeds and nuts to consumers and people who deal with nutrition. Also, results are expected to increase the economic value of sacha inchi residue as a source of diversification of food products in Taiwan.

## 1. Introduction

Sacha inchi (Plukenetia volubilis) is a source of quality commercial oil. In Taiwan, its commercial production commenced in 2015, and the area has reached 1200 ha [1]. Sacha inchi is also known by different names such as sacha peanut, sacha maní, incha peanut, maní del Inca, incha inchi, supua, ticazo, and mountain peanut [2]. Oil extraction from sacha inchi is achieved mechanically with an oil press machine, which results in sacha inchi residue that remains unutilized. Also, its nutritional value has not been widely investigated in Taiwan. The oil extraction process, seed processing (e.g., roasting before extraction), growth process, and genetics influence oil contents in sacha inchi [3]. Also, seeds and nuts have slightly different nutritional levels [4].

Sacha inchi has been known to be rich in unsaturated fatty acids [3]. The uniqueness of sacha inchi is that it contains 93% fatty acids, most of which are essential oils, which are important for health. The levels of these essential oils are closely related to the crop zone [5]. Essential fatty acids in the form of omega 3 and omega 6 contained in sacha inchi oil are 50.5% and 34.1%, respectively, while the protein content in inchi is 22-30%. In Peru, in addition to being a raw material for quality oil, sacha inchi can be roasted, salted, or mixed with chocolate, and used as ingredients for making butter [6]. Sacha inchi commercial oil has a high content of tocopherol, omega 3, omega 6, omega 9, and palmitate. Tocopherol is an antioxidant that can stabilize oxidation reactions in oil [7]. Sacha inchi also contains stearic acid, phenyl alcohol, flavonoids, and sterols. Consuming 50 g of sacha inchi can significantly lower triglyceride levels. Sacha inchi oil has an unpleasant taste, but over time, it can be accepted by consumers [8]. Sacha inchi contains bioactive compounds that are beneficial for health. Commercial sacha inchi oil is classified as extra virgin oil and virgin oil, and its oxidative stability is low so it should not be consumed hot [9]. The lack of consumer and industry knowledge about the content and benefits of sacha inchi makes this plant not optimally utilized and even ignored. This plant has the potential to be a source of nutritious food to overcome food scarcity [10].

A grain, seed, nut, and bean are related but different from a botanical standpoint. A grain is a small edible fruit harvested from grassy crops. A seed is defined as an embryonic plant covered in a “seed coat.” A nut is a tree seed contained in a hard shell [11]. Bean is a specific variant of seed that grows in a pod that splits down both sides. Peanut is a leguminous plant. However, it is classified as a nut [4]. Vegetable oil is classified into two types based on its function: edible and nonedible. Edible oils are generally used in the food and medicine industry, while nonedible oils are used as raw materials for biodiesel [12].

Daily nutrition can be obtained by consuming seeds and nuts, but not all nuts can be consumed directly, such as sacha inchi and candlenuts. Raw candlenuts contain toxalbumin, which can cause blood clots [13]. The benefits of vegetable oil depend on its type of fatty acid. There are five commonly fatty acids methyl ester found in vegetable oils: palmitate, stearate, omega 9, omega 6, and omega 3 [14]. The oil content depends on the type of seed and nut. Seeds and nuts are sources of protein and carbohydrates. Sweet and bitter taste depends on their sugar content. In general, the sweetness of seeds and nuts is lower than fruits. The total sugar in peanuts depends on the variety and ranges from 19% to 21% [15]. The type of sugar that is closely related to insulin is glucose. Diabetic patients are advised to be careful about their daily sugar consumption [16]. Omega 3 is a potential supplement for type 2 diabetes patients [17].

Near-infrared (NIR) spectroscopy is an effective tool for testing food quality. NIR is nondestructive, efficient, and does not require a solvent. Another feature of the NIR is that the analysis can be performed in a shorter time, and the method can be repeatedly used after optimization [18]. NIR can be used for qualitative and quantitative analysis of liquid and solid samples [19]. Several molecular bonds that respond to NIR are −OH, −CH, −CO, and −NH [20]. Chemical bonds inside the food can absorb energy in the NIR region; therefore, this tool can be used to observe the behavior of chemical bonds in the food. One of the pretreatment processes is standard normal variate transformation (SNV), which reduces noise, baseline drift, and spectral scattering. The following process is to correlate the spectra with the results of laboratory measurements to obtain NIR predictions. The models commonly used are partial least squares (PLS) and principal component regression (PCR) [18, 21, 22]. The application of NIR for seeds and nuts has been carried out by several researchers, using walnut kernels [22], pine (Pinus koraiensis) seeds [23], cotton seeds [19], and almonds [24]. The NIR method can be applied for drying, peeling, and processing peanuts factories [20].

Characterization of functional groups has been done by nuclear magnetic resonance (NMR) spectroscopy. NMR method has advantages such as a shorter time for sample preparation, a small amount of solvent, and a shorter duration for analysis. Our unpublished research found that the correlation of fatty acid levels using NMR and gas chromatography flame ion detector (GC-FID) has a Pearson's *R*^2^ of 0.983. The correlation between the two methods is strong and positive for determining the fatty acids from sacha inchi at different maturity levels, but NMR is greener than GC-FID. Another nutrient found in vegetable and animal oils is resveratrol. Resveratrol functions as an antioxidant [25, 26]. Other benefits of resveratrol are antitumor [25], anticardiovascular disease, antibacterial, anti-inflammatory, antiaging [27], anticancer, antiobesity [28, 29], and potential as antidiabetic [30]. Resveratrol has been found in peanuts with levels 0.022-1.792 ppb. Its levels can be reduced due to the roasting process. The resveratrol levels also depend on genetic and cultivation factors [31]. Resveratrol in peanut is 0.71 ppb [32]. The heating process reduces resveratrol levels in cashew nuts, and the levels were reduced from 1.4 mg/100 g to 0.9 mg/100 g by the microwave heating range of 180-720 W [33]. LC-MS/MS is a sensitive method for a small module weight analysis. This method has been applied for detection, identification, and quantification [34]. Unlike oil, sugar is polar and nonvolatile; hence, analysis is performed using high-performance liquid chromatography (HPLC). Analysis of three types of sugars has been carried out using the HPLC with evaporative light scattering detection (ELSD) method [35–40]. ELSD detector has succeeded in detecting 13 types of sugars in foods [41].

Based on the background, it is necessary to analyze the nutritional levels of various seeds and nuts, especially edible seeds and nuts with high consumption compared with sacha inchi residue. The methods used are NIR spectroscopy, NMR spectroscopy, LC-MS/MS, and HPLC-ELSD. Therefore, this study aims (a) to determine the oil, moisture, ash, protein, carbohydrate, type of fatty acid, resveratrol, and type of sugars and (b) to determine the model to predict the five major nutrients. The results of this study are expected to provide information on nutrient levels in seeds and nuts to consumers and people who deal with nutrition. Information about the nutrients in the sacha inchi residue is expected to increase the economic value of sacha inchi residue, becoming a reference for the diversification of food products in Taiwan.

## 2. Materials and Methods

### 2.1. Materials and Equipment

Peanut, sesame, sunflower, and black bean were obtained from the traditional market in Taiwan. Candlenut was obtained from the Indonesian market in Taiwan, and sacha inchi residue was collected from Shetao Farm, Changhua, Taiwan. The reagents used were n-hexane high grade (CAS 110543), methanol 99.5% (Merck), acetonitrile, hydrogen peroxide, sodium hydroxide, sulfuric acid, dichloroform (Sigma Aldrich), and tetramethylsilane (Sigma Aldrich), and resveratrol CAS 501360 (228.24 g/mol). Soxtec™ 8000 extraction unit (FOSS), Kjeltec™ 8100 distillation unit (FOSS), oven (DOS45), oven TENDER FCS Shinho, analytical balance (AND GR-200), moisture analyzer (AND MX-50 JASCO), ultrasonic vibration, HPLC Shimadzu Prominence-i LC-2030C Plus with SEDEX LT-ELSD Model 85LT, NMR Spectrometer Bruker Avance 500 MHz complete with UltraShield™ superconducting magnet, constant temperature system B-VT 3000, SpectroSpin 11.7 Tesla, standard diameter 54 mm, Probe 5.0 mm and topspin 2.1 software for NMR data processing, Microsoft excels, Origin software for ANOVA one-way analysis. *p* values <0.05 are considered statistically significant different, and *p* values >0.05 are considered not statistically significant different.

### 2.2. Near-Infrared Procedure

**Figure 1:**
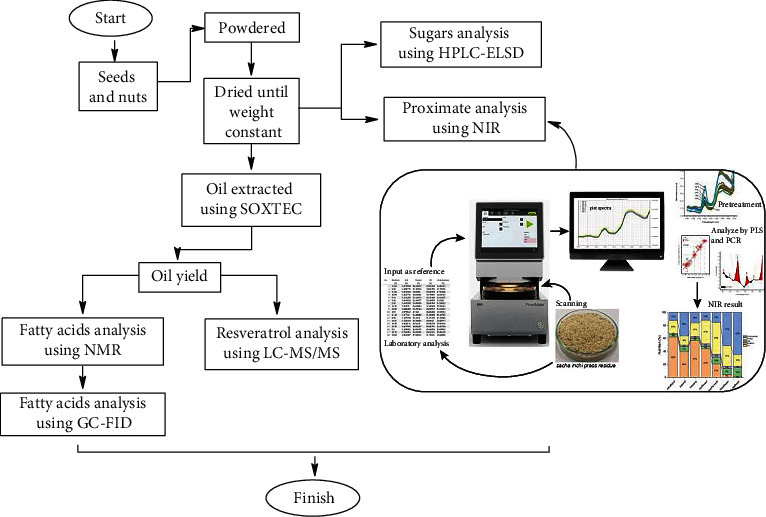
Flowchart nutrient analysis.

Dry seeds and nuts of different plant species were ground separately. A total of 15 g of each sample were taken into separate Petri dishes, and these were treated with light from top to bottom. NIR spectroscopy wavelength range of 900–1700 nm, the bandwidth of 7 nm, scanning speed 100/second, and analysis 15 seconds. Scanning of each sample was done with 18 repetitions. The overall nutritional analysis is shown in Figure 1.

#### 2.2.1. Oil Content Analysis Procedure

Samples of candlenut, peanut, sesame, sunflower, sacha inchi residue, black bean, and red bean were powdered separately, dried at 50°C to constant weight, and then stored in a closed container. Determination of total oil in the laboratory was done with Soxtec. The extraction conditions were boiling time 40 min, hexane 50 mL, and temperature 135°C. The oil extraction procedure was carried out using 3.0 g of powdered sample. Oil content analysis was performed for each sample with 18 repetitions. In this study, the seeds and nuts were ground roughly. The grinding process only takes 3 to 5 min. Then, put in the oven for up to 48 h at 50°C, to obtain optimal oil content, especially for sacha inchi residue. In addition, it aims to prevent the growth of microorganisms that can reduce the quality of seeds and nuts during storage.

#### 2.2.2. Moisture Content Analysis Procedure

Moisture analysis was carried out on the sample before being used for other analyzes. A total of 2.0 g of sample was taken into an aluminum plate. The moisture analyzer was set to 125°C and then waited until it showed the percentage of moisture. Moisture content analysis in each sample was performed with 18 repetitions.

#### 2.2.3. Ash Content Analysis Procedure

The porcelain cup was weighed, and 2.0 g of the sample was added and then kept in the TENDER oven. The sample was heated for 6 h at 600°C, and the sample was cooled in a desiccator and then weighed. The percent of ash was obtained from the difference between the initial weight and the final weight of the porcelain cup. Ash content analysis in each sample was performed with 18 repetitions.

#### 2.2.4. Protein Content Analysis Procedure

Each sample was weighed 2.0 g and added with 10 mL concentrated sulfuric acid and 0.5 g accelerating agent. In this study, we used an accelerating agent in the form of solid copper sulfate and potassium sulfate in a ratio of 1 : 4. The mixture was cooled at room temperature and added with 2 mL of 30% hydrogen peroxide. The sample was digested at 400°C for 4 h and then cooled. The sample was distilled using Kjeltec to convert ammonium to ammonia using an alkaline solution. The distillate was collected in an Erlenmeyer flask containing 25 mL of 0.05 N H_2_SO_4_ and 2 drops of indicators and then titrated with 0.05 N NaOH. The total protein content was calculated using Equation (1). Protein content analysis was performed for each sample with 18 repetitions.

(1) $$\begin{array}{c} \text{nitrogen }\left(\%\right)=\frac{\left(\left(\text{m}{\text{L}}_{\text{sample}}-\text{m}{\text{L}}_{\text{blank}}\right)\times N\times 14.007\times 100\right)}{\text{m}{\text{g}}_{\text{sample}}}. \end{array}$$

#### 2.2.5. Carbohydrate Content Analysis Procedure

The carbohydrate content can be calculated using the following equation and performed in each sample with 18 repetitions.

(2) $$\begin{array}{c} \text{carbohydrate }\left(\%\right)=100-\left(\text{moisture }\left(\%\right)+\text{protein }\left(\%\right)+\text{oil }\left(\%\right)+\text{ash }\left(\%\right)\right). \end{array}$$

### 2.3. Analysis for Types of Fatty Acids Using ^1^H NMR

100 *μ*L oil was mixed with 600 *μ*L of d-chloroform in an Eppendorf tube. Homogenization was carried out for 5 min; then, a drop of TMS was added and homogenized again. The mixture was transferred to an NMR tube and subjected to analysis using ^1^H NMR.

### 2.4. Analysis for Resveratrol Extract Using LC-MS/MS

200 Mg of cotton fiber was put into 10-mL syringe. The cotton was activated using 2 mL of hexane and then pumped. Cotton was soaked in 3 mL oil in hexane (1 : 3) and then pumped. The cotton was rinsed using 2 mL of ethanol. The extract was collected for analysis using LC-MS/MS. LC-MS/MS set up flow rate 0.350 mL/min, mobile phase deionized water and methanol (8 : 2), sample injection 2 *μ*L, gas temperature 300°C, and nebulizer 45 psi.

### 2.5. Analysis for Types of Sugars Using HPLC-ELSD

1.0 g of sample was mixed with 10 mL of deionized water, vortexed for 5 min until homogeneous, and then put into an ultrasonic vibrator for 60 min. Each standard sugar (xylose, fructose, sorbitol, galactose, glucose, sucrose, lactose, and maltose) was weighed (10 mg) and dissolved into 1 mL of deionized water. The standard solution was made from a sugar mixture of 100-1200 ppm and then analyzed using HPLC-ELSD. Mobile phase deionized water and acetonitrile, HPLC column temperature 60°C, volume injection 5 *μ*L with flow rate 700 *μ*L/min, wavelength 254 nm, ELSD system temperature 50°C, gas pressure 35 psi, the value of gain 9.0 with pressure control 8.5 MPa.

## 3. Results and Discussion

### 3.1. Near-Infrared

**Figure 2:**
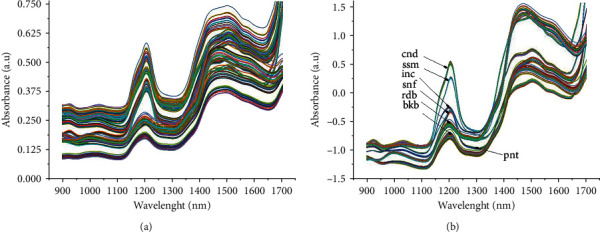
Smoothing spectra by Savitsky-Golay (a) and SNV pretreatment spectra (b) of candlenut (cnd), peanut (pnt), sesame (ssm), sunflower (snf), sacha inchi residue (inc), black bean (bkb), and red bean (rdb).

Standard normal variate transformation (SNV) pretreated spectra were not significantly different from smoothing spectra (Figure 2). SNV was performed to reduce baseline drift and scattering spectra [22]. The spectral pattern of each seed and nut sample appeared the same. NIR can absorb light from bonding vibrations of C−H, O−H, and N−H. Inorganic compounds are not detected in the wavelength range of 780–2500 nm [42]. The original spectra are composed of a lot of noise and aberrations, so pretreatment is necessary. Several pretreatment options that can be used are first derivative, second derivative, multiple scattering correction, wavelet transform, standard normal variate, Savitsky Golay, and Norris derivative [42]. Savitsky Golay is commonly used to reduce noise [43]. There is absorption at the wavelength 958–1071 nm, 1124–1260 nm, and 1321–1653 nm. Absorption at 1400–1440 nm and 1900–1950 nm was identical to moisture inside the food [44]. Compared to all functional groups, the −OH bond vibration has a stronger absorbance than other functional groups at the peak of 1463 nm. The sample seeds and nuts contain a nutrient mixture such as oil, moisture, mineral, protein, and carbohydrates. NIR interpretation data is presented in each subsection of nutrient analysis.

The accuracy of the prediction of chemical composition content can be seen from the validation. The values are based on standard calibration error (SEC) and standard error prediction (SEP) [44]. To improve the accuracy of the prediction numbers, cross-validation was carried out, and the samples were selected randomly. The reliability and stability of the prediction model can be seen from the RMSECV (root mean standard error of cross-validation) and RMSEP (root mean square error of prediction) [23]. The external validation method has been applied to analyze oil using NIR [45]. In this experiment, validation is done through cross-validation. The total number of samples is 140. A total of 20 samples from the total number were randomly selected for cross-validation.

Calculating a confidence interval in Table 1 is shown as SEC (standard error of calibration), SECV (standard error of cross-validation), SEP (standard error of prediction), and RPD (residual predictive deviation or ratio standard error prediction to deviation). RPD is obtained from the standard deviation divided by the standard error prediction [44]. These parameters are used as a reference for choosing the right method for determining oil, moisture, ash, protein, and carbohydrates.

#### 3.1.1. Oil Analysis

**Figure 3:**
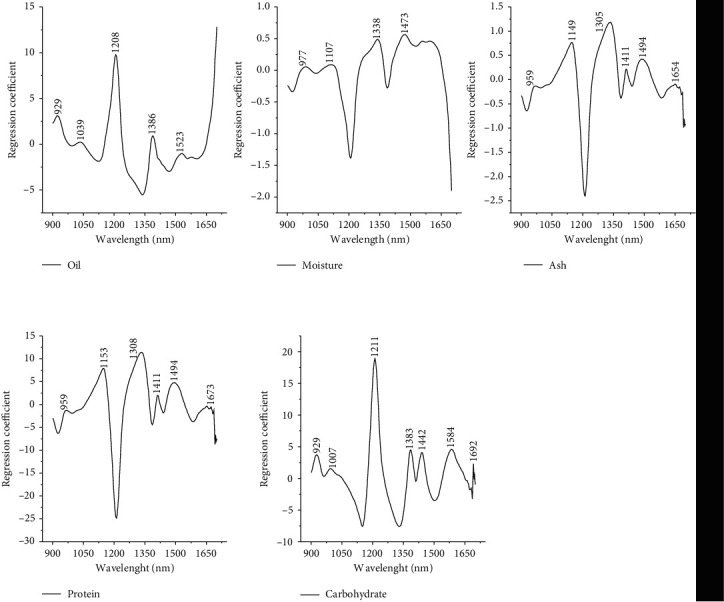
The regression coefficient of nutrients by NIR spectroscopy.

The oil extracted from each sample has differences in viscosity and color. Peanut has a viscous oil and quickly solidifies at room temperature among all samples. Oil viscosity is affected by temperature and has an impact on FAMEs. Heating at 40°C causes the viscosity of FAMEs to decrease, and the value is lower than in vegetable oil. Waste oil has a higher viscosity than fresh oil. A reasonable oil requirement for a lubricant has a practically stable viscosity concerning temperature changes. Information about the viscosity of vegetable oils is vital for the lubricating oil industry [46]. The increase in viscosity causes an increase in the number of double bonds in the oil [47]. Oil viscosity is correlated with oil mass. High viscosity indicates that the oil mass gets heavier at the same volume condition, and the distribution coefficient is slower. Our previous research showed that the slowest distribution coefficient pattern on soybean oil had been repeatedly heated. Based on this, it can be seen that peanut oil is not suitable as a source of lubricating oil because of its unstable viscosity when heated. The striking color difference comes from black bean oil. The color looks dark green, while the oil from other seeds and nuts is brownish yellow. The black bean coat contains natural pigments in the form of anthocyanin compounds and other phenolic compounds. 10% by weight of beans is the coat, and the content of phenolic compounds in the coat is higher than in the beans [48]. The analysis of functional groups that contribute to linear regression can be seen in the regression coefficient. Lipids have absorptions at 1500, 1720, 1760, and 2350 nm [49, 50]. The vibrations bond of −CH, −CH_2_, and −CH_3_ have absorptions at 1053, 1415, 1620–1765, and 2310–2323 nm [44]. The black cumin seeds oil results showed a peak at 1371 belonging to −CH_2_ bending vibration at the triglyceride group, while 1095 and 1237 belonged to stretching vibration −CO at the triglyceride ester group [51]. The −CH stretching vibration on the fatty acid structure (R − C=O − OH) also has absorption at 1215, 1395, 1725, 1765, 2200, and 2310 nm. Thus, the oil regression coefficient shows that the peaks of 929 nm, 1039 nm, 1208 nm, 1386 nm, and 1700 nm (Figure 3) represent oil chemical structure.

Based on the data in Table 1, the determination of oil content using Soxtec has an *R*^2^*C* of 0.998. Using NIR, determining oil levels is considered an excellent classification compared to other nutrients. This can be seen from the RPD, which is more than 13. Both PLS and PCR models can predict oil content in seeds and nuts with *R*^2^*P* 0.997. Compared to PLS, PCR has a smaller SEP of 1.690. This shows that the PCR method is better than PLS for predicting the oil content in seeds and nuts.  Table 2 shows that the highest oil content is found in candlenut, followed by sesame, sunflower, peanut, sacha inchi residue, black bean, and red bean, respectively. Candlenut from Indonesia has been reported to contain 61.4% of oil [52]. Sesame has high-quality oil and is called the queen of oilseeds [53]. Our previous studies used sacha inchi, which produced an oil content of 51.71% [14]. This shows that the pressing process cannot extract all oil, and extraction using Soxtec can take back the oil in the sacha inchi 20.941%. The difference between the extraction method by mechanical (pressing) and the Soxtec method is the presence of solvents. Although Soxtec is efficient and works automatically, the solvent residue is toxic if consumed directly. Based on the *p* value for oil content, there was a significant difference in oil content with all seeds and nuts. High-grade oil is a good source for industrial purposes [54].

#### 3.1.2. Moisture Analysis

Moisture in food is related to mineral ions, organic monomers, and polymers linked through hydrogen bonds. The quality of photosynthesis in the sacha inchi plant is also affected by waterlogging [1]. In addition, the role of moisture also involves the food fermentation process [55]. The information on the moisture content is vital for the shelf life and transportation of food materials. Water is a medium for the growth of fungi and bacteria that accelerates decay. The moisture content of nuts and seeds should be reduced to 10.5% before these are marketed [56].

The ability of the material to absorb water molecules can increase the standard error of prediction [57]. Moisture analysis results show the opposite regression coefficient pattern with oil. Some peaks appear at 977 nm, 1107 nm, 1338 nm, and 1473 nm (Figure 3). The presence of water in the sample can be seen based on the −OH stretching vibration, which has an absorption at 1400 nm [18]. The wavelength close to 1480 and 1900 nm is a selective ratio for water in the PLS prediction model [49].

In our study, there are four peaks in 1st derivative spectra of NIR, at 970 nm, 1190 nm, 1450 nm, and 1940 nm belonging to −OH stretching, a combination of −OH starching and bending, and −OH bending, and the strong peaks are at 1400–1440 nm and 1900–1950 nm similar to an earlier study [55]. Oliveira and Franca also reported the same results. 1450 nm and 1950 nm belong to the vibration of the −OH bond in water molecules [21]. The moisture regression coefficient is similar to oil but the opposite. This pattern shows that the higher the moisture content, the lower the oil content. NIR can predict moisture content excellently. Both PLS and PCR can be used as references for appropriate statistical methods to predict moisture content, with *R*^2^*C* of 0.989 and RPD of 6.678 and 6.702, respectively. The highest moisture content was found in the sample of red bean, followed by sacha inchi residue, black bean, peanut, candlenut, sesame, and sunflower, respectively (Table 2). The *p* value showed no significant difference in moisture content between sesame and sunflower.

#### 3.1.3. Ash Analysis

The regression coefficient shows positive values at wavelengths 1149 nm, 1305 nm, 1411 nm, and 1494 nm (Figure 3). The wavelength that appears in the analysis of the ash content is 929.5–1583.5 nm [57]. The best statistical method for predicting ash content is PLS with an *R*^2^*C* of 0.961 and RPD of 3.245. Ash is composed of minerals in seeds and nuts. The high mineral content can be seen from the amount of ash content [40]. Our study found the highest ash content in sacha inchi residue, followed by sunflower, black bean, sesame, red bean, peanut, and candlenut, respectively (Table 2).

Sacha inchi has 2.70–6.46 g/100 g ash content. Ash is composed of calcium, phosphorus, sodium, potassium, magnesium, copper, iron, manganese, and zinc [10]. Black bean has been reported to contain 6.5 mg iron, 4 mg zinc, and 1 mg copper [58]. Sesame is a source of minerals such as iron, calcium, iodine, and zinc [53]. Red bean contains minerals such as calcium, nickel, zinc, copper, iron, chromium, sodium, potassium, phosphorus, and magnesium [54]. Peanuts contain potassium, calcium, phosphorus, iron, zinc, copper, manganese, cadmium, and lead [32]. Candlenut is composed of manganese, calcium, magnesium, phosphorus, and potassium [59]. Candlenuts also contain iron, zinc, copper, and selenium [13]. Based on the *p* value, the ash content was not significantly different between black bean with sunflower and red bean with sesame. NIR predicted the ash content with excellent prediction based on the RPD value.

#### 3.1.4. Protein Analysis

Protein molecules are composed of amino acid monomers linked together by peptide covalent bonds. At 1200 and 1940 nm, absorption belongs to protein, amino acid, and moisture [49, 50]. The absorption in the range of 1400 and 1600, specifically 1530 nm, belongs to the −NH functional group of proteins in almonds [24]. The regression coefficient shows peaks at wavelengths 1153 nm, 1308 nm, 1411 nm, 1494 nm, and 1673 nm (Figure 3). The peak pattern of protein regression coefficients is similar to the ash pattern, but protein has a regression coefficient value close to 6-12 times higher than the ash regression coefficient.  Table 1 shows that the appropriate statistical method for predicting protein content is PLS, with *R*^2^*C* 0.950 and RPD 2.886.

RPD >3 means were excellent, in the range 2–3 means very reliable predictions, and range 2.5–5 means limited prediction, while <1.5 means unreliable prediction [19]. Predicting protein content using PLS and PCR is very reliable compared with oil, moisture, ash, and carbohydrate content. One of the reasons is the conventional titration involvement in determining protein content or human error. Samples with high protein content are sacha inchi residue, followed by black bean, sunflower, sesame, candlenut, red bean, and peanut, respectively (Table 2). The *p* value showed no significant difference in protein content between sesame with candlenut, black bean with sunflower, and red bean with candlenut and sesame. Sacha inchi has a protein composition of albumins, globulins, glutelins, and prolamins [10].

Sunflower has proteins such as alanine, glycine, glutamic acid, leucine, and aspartic acid [60]. The temperature at 25°C and 35°C with 17% moisture can affect mold development, which influences internal hydrophobic sites, and degradation of proteins. Black beans and red beans contain globulins and albumins [61]. The proteins in candlenuts are glutamic acid, leucine-isoleucine, valine, tyrosine, lysine, methionine, phenylalanine, alanine, serine, cysteine, threonine, proline, and toxalbumin. Toxalbumin is a toxic protein that can cause blood clots and inhibit the synthesis of other proteins [13]. Peanut is an important source of protein in many countries [62]. The protein composition in peanuts is glycine, lysine, glutamic acid, asphaltic acid, and methionine [63].

#### 3.1.5. Carbohydrate Analysis

Carbohydrates have a peak of 2350 nm [49, 50]. The wavelengths at 835 nm, 911 nm, 948–962 nm, and 999–1003 nm are sensitive to reducing sugars in NIR spectra. Changes in sugar structure due to fermentation can be observed in the 1650–1750 nm. This is also an indication of the formation of tannins and phenolic compounds [21].

Figure 3 shows the wavelengths with a positive regression coefficient of 929 nm, 1007 nm, 1211 nm, 1442 nm, 1584 nm, and 1692 nm. The characteristic of the carbohydrate functional group is stretching vibration −CO appearance [24]. The carbohydrate content prediction model is PLS because it has R^2^C 0.980 and RPD 4.666 (Table 1). This shows that the prediction of carbohydrates in the sample is excellent. The samples with the highest carbohydrate content are red bean, followed by black bean, peanut, sunflower, sesame, sacha inchi residue, and candlenut, respectively (Table 2). The *p* value result showed no significant difference in carbohydrate content between sacha inchi residue with candlenut and sesame. A high level of carbohydrates means good energy sources for feeds supplement [54]. Also, carbohydrate is a major energy source for plants [64]. Carbohydrate synthesis correlates with plants' chlorophyll, water, and carbonic gas function [65].

### 3.2. Fatty Acid Analysis Using ^1^H NMR

Fatty acid analysis generally uses GC. Separation is based on the difference in retention time. FAME is retained longer in the stationary phase and has a longer retention time. This causes the analysis using GC to be more accurate. Compared with GC, NMR is simple and greener and takes a shorter duration for sample preparation and analysis. Also, the method does not require many reagents and can be used for quantitative and qualitative analysis based on peak integral ratios using Equations (3)–(6) [66]. Peak A belongs to the methyl group of FA, while peak B belongs to the acyl omega 3. Peak C belongs to the allylic functional group, Peak D belongs to methyl bonded to the carboxyl functional group, and peak E belongs to the proton in UFA.

(3) $$\begin{array}{c} \text{omega }3\;\left(\%\right)=100\left[\frac{B}{A+B}\right], \end{array}$$

(4) $$\begin{array}{c} \text{omega }6\;\left(\%\right)=100\left[\left(\frac{E}{D}\right)-2\left(\frac{B}{A+B}\right)\right], \end{array}$$

(5) $$\begin{array}{c} \text{omega }9\;\left(\%\right)=100\left[\left(\frac{C}{2D}\right)-\left(\frac{E}{D}\right)+\left(\frac{B}{A+B}\right)\right], \end{array}$$

(6) $$\begin{array}{c} \text{SFA }\left(\%\right)=100\left[1-\left(\frac{C}{2D}\right)\right]. \end{array}$$

Based on the NMR result, omega 3 levels are sequentially found in sacha inchi residue, candlenut, and black beans. Omega 6 levels are found in sunflower, black bean, sesame, candlenut, peanut, and sacha inchi residue. Omega 6 and omega 3 are PUFAs. The ratio of omega 6 and omega 3 (1 : 1) in the oil has an optimal effect on health [3]. Samples that have this ratio are sacha inchi residue (0.767), candlenut (1.804), and black bean (4.443). Our unpublished work found that omega 3 can be reduced by heating. Omega 3 was found in soybean oil and reduced by heating at 190°C.

Heating can change the geometrical double bond formation into trans-fatty acids. A frying temperature of 180°C triggers the reaction of oxygen with unsaturated fatty acids into hydrogen peroxide [67, 68]. Oils rich in omega 3 are sensitive to heat, oxygen, and light [69]. NMR spectra showed that there was a decrease in the proton signal intensity belonging to omega 3 followed by a decrease in the proton signal omega 6. This occurred in samples heated at a frying temperature [69].

On the other hand, the other factor affecting fatty acid content is the fruit maturity stages. The mature sacha inchi produce high oil content and more fatty acids compared with immature. The current study's correlation between NMR and GC FID results shows a positive linear regression of *R*^2^ 0.933. Compared with the NMR result, the GC-FID result showed that all samples contain various fatty acids, including omega 3, omega 6, and omega 6.

### 3.3. Resveratrol Extract Analysis Using LC-MS/MS

Resveratrol is a polyphenol compound with two aromatic rings [70]. Resveratrol is rich in electrons to act as a ligand for proteins. The 3',5' hydrogen of trans-resveratrol is the active site of trans-resveratrol. Cis and trans-resveratrol depend on olefinic arrangement structure [34]. Concerning polarity, resveratrol is polar, while oil is nonpolar. Resveratrol dissolves in methanol, ethanol, and DMSO. In our study, sample preparation used cotton to absorb resveratrol from seeds and nut oil. Cotton, hexane, and ethanol have been used to extract resveratrol [71]. Resveratrol was tested using LC-MS/MS. One of the advantages of MS is that it can identify nonisobar compounds without involving radiolabeled regents. Radiolabeled regents are usually used to separate ionic fragments in the conventional method. The level of fragmented molecules is based on the relative intensity [72]. The combination of chromatography and mass spectroscopy can detect the separation of components based on retention time and provide information about organic molecules' structure [73].

**Figure 4:**
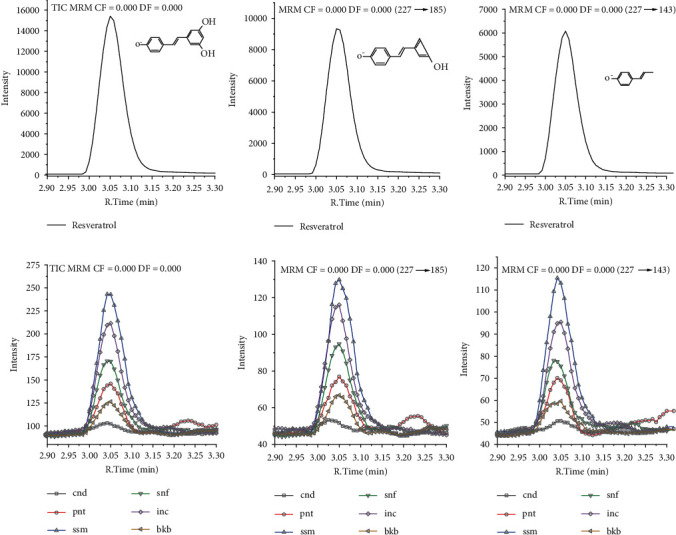
LC-MS/MS spectra of candlenut (cnd), peanut (pnt), sesame (ssm), sunflower (snf), sacha inchi residue (inc), black bean (bkb), and fragment ion of resveratrol.

Matrix effects have been reported to affect the sensitivity, ionization efficiency, and retention time of resveratrol assays in plasma rats using HLPC-MS/MS [74]. MS/MS has two mass analyzers, also known as tandem mass spectrometry. Mass analyzer 1 identifies ion precursors, while mass analyzer II identifies products in ionic fragments. The mass of the ionic fragment is related to the molecular mass of the analyte, expressed in units of m/z [18]. The ions fragment 185 m/z and 143 m/z of resveratrol has been found from rat urine [75], sorghum [38], and pure trans-resveratrol [76]. Resveratrol's hydrogen is very close to the liposome's lipid chain [70, 77]. In this study, LC-MS/MS detects standard resveratrol with a retention time of 3.050 min, with a precursor ion mass of 227 m/z, and the resulting ionic fragments have masses of 143 m/z and 185 m/z. Peaks of these ion fragments were also found in all samples of seeds and nuts, with different intensities (Figure 4.). The LC-MS/MS results showed that the highest levels of resveratrol were in sesame, followed by sacha inchi residue, sunflower, peanut, black bean, and candlenut, with a total average of 1.815 *μ*g/100 g oil. The linear calibration curve has *R*^2^ 0.998. An increase in the resveratrol concentration affects the increased peak area of LC-MS/MS at 3.050 min. Maximum resveratrol levels of 2.746 *μ*g/100 g oil were found in sesame (Table 2). Sesame has been reported to contain another phenolic compound, namely, sesamol. Pure sesamol and pure resveratrol both have antioxidant activity when tested by the DPPH assay method. Resveratrol, sesamol, sunflower oil, and sesame oil have the potential for antioxidant activity, respectively [25]. The *p* value result showed no significant difference in resveratrol between sacha inchi residue with sesame, black bean with candlenut, and peanut.

### 3.4. Sugar Analysis Using HPLC-ELSD

Carbohydrate values correlate with sugar content in the sample. Information on sugar content is crucial; hence, it is shown in nutrition labels on food packaging [40]. The total monosaccharides and disaccharides are the total sugars in the diet. There are sugar alcohol terms such as xylitol, sorbitol, and maltitol. Sugar alcohol is used as a substitute for sugar, but excessive consumption can interfere with the digestive system and blood sugar levels [41]. The function of sugar and sugar alcohol in plants is to maintain ionic and osmotic balance [35]. Fructose, glucose, and galactose are called reducing sugars. Based on the level of sweetness, the sweetest sugars are fructose, sucrose, and glucose, respectively [78]. In this study, the identification of eight types of sugars in the samples was also carried out. The sugar extraction involves deionized water. This is because sugar is a substance that is soluble in water. Cyclic monosaccharide structures can open when dissolved in water. This condition constantly occurs at high speed. The open form of cyclic monosaccharides causes the carbonyl group to interact with other molecules easily.

Based on the shape of the chain, amylose has a linear chain, while cellulose is in the form of a helix. Suitable solvents for cellulose polymer derivatives/amylose polymer derivatives are polar solvents or with alcohol mixtures. The addition of a solvent is vital because the polymer cannot be chemically bonded to the silica (as a stationary phase) directly. Under standard conditions, phase chromatography, the sample is hydrophilic and can be separated with a polar solvent as a mobile phase, such as methanol, water, acetonitrile, or tetrahydrofuran). Meanwhile, the stationary phase is nonpolar. The higher the polarity of the mobile phase, the shorter the elution process. Partition and adsorption processes can overlap [18].

**Figure 5:**
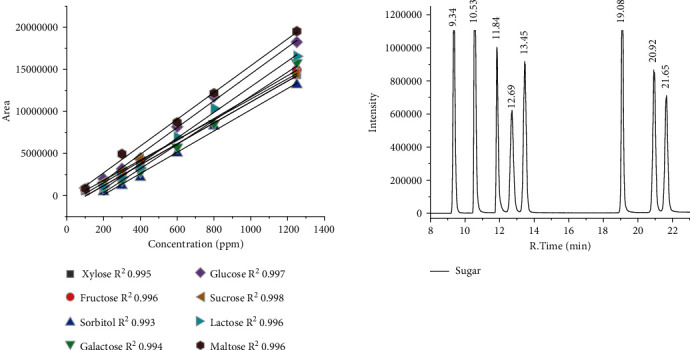
Calibration curve and chromatogram of sugar standard.

The type of sugar was analyzed using HPLC-ELSD. The ELSD detector can respond to nonvolatile solutes, while the mobile phase is limited to only volatile ones. The sample mixture and the mobile phase are converted into mist in a nebulizer and then scattered radiation at the right angle [18]. In this study, the sugars identified were xylose, fructose, sorbitol, galactose, glucose, sucrose, lactose, and maltose. The retention time of each sugar was 9.34 min, 10.53 min, 11.84 min, 12.69 min, 13.45 min, 19.08 min, 20.92 min, and 21.65 min, respectively (Figure 5). This shows the phenomenon of interaction time between the type of sugar and the stationary phase, xylose < fructose < sorbitol < galactose < glucose < sucrose < lactose < maltose. Xylose, fructose, galactose, and glucose are monosaccharides based on the molecule size. Sucrose, lactose, and maltose are disaccharides. Clemens et al. said that sorbitol is sugar alcohol from glucose [79]. This indicates that monosaccharides separate faster than disaccharides.

A total of eight sugar content was found in sacha inchi residue, sunflower, sesame, candlenut, peanuts, and black bean in 913–5055 mg/100 g flour. Among the eight types of sugars identified, sucrose was the primary sugar found in sacha inchi residue, sunflower, sesame, candlenut, black bean, and peanut, respectively. The average value of sucrose was 1352 mg/100 g. There was no significant difference in sucrose between sesame with candlenut, sacha inchi residue with sunflower, and black bean with peanut. Compared with all samples, peanuts are rich in xylose. *p* value showed a significant difference in xylose between peanuts with all samples. Fructose is mainly found in sacha inchi residue and peanuts. *p* value showed a significant difference in fructose between sacha inchi residue in all samples. Compared with all samples, sesame has high amounts of sorbitol. Between sacha inchi residue and candlenut also showed *p* values <0.05, which were considered significantly different in sorbitol content. Galactose is mainly found in peanuts, and the concentration is significantly different in all samples. A high glucose level was found in sacha inchi residue and peanuts. Both sacha inchi residue and peanut have no significant difference in glucose level. *p* value showed significantly different glucose contents between sacha inchi residue with candlenut, sesame, sunflower, and black bean. In contrast, glucose in peanuts is significantly different from sesame, sunflower, and black bean. The lactose concentration ranges from 131.680 to 179.480 mg/100 g. The *p* values >0.05 were considered not significantly different in lactose and maltose content from all samples.

## 4. Conclusions

Comparing nutrition in sacha inchi residue and various edible seeds and nuts has been carried out using spectroscopy and chromatography studies. The results showed that each sample had varying levels of the five major nutrients. NIR spectroscopy can efficiently predict oil, moisture, carbohydrate, and ash levels, while protein content prediction is reliable. Prediction results using NIR spectroscopy show that the highest oil content is found in candlenuts. Compared with the five major nutrients, there is a significant difference in oil content between all samples. The highest moisture, ash, and protein levels in the sacha inchi residue were found. NMR spectroscopy results showed that all samples were rich in PUFAs in omega 3 and omega 6, respectively. PUFAs were optimum in sacha inchi residue and sunflower. While omega 9 was found in peanuts, LC-MS/MS analysis showed that the maximum resveratrol was found in sesame though it is present in all samples in small amounts. HPLC-ELSD analysis showed that samples contained high sucrose levels, except for peanuts and black beans. The highest sucrose level was found in sacha inchi residue, sunflower, sesame, candlenut, black bean, and peanut. Peanut is rich in glucose, while the black bean is rich in galactose. Based on the *p* value, there were no significant differences in lactose and maltose in all samples. Consumers often ignore the information on nutritional levels in food, even though it is important to control the amount of nutrient intake needed by the body. Given that excess or lack of nutritional intake can harm the body.

## Figures and Tables

**Table 1:** Calibration, cross-validation, and prediction in different models by NIR spectroscopy.

Models	Nutrition	Calibration				Cross-validation		Prediction			RPD
		RMSEC	SEC	*R* ^2^ *C*	RMSECV	SECV	*R* ^2^CV	RMSEP	*R* ^2^ *P*	SEP	
PLS	Oil	1.611	1.656	0.998	1.540	1.533	0.998	1.733	0.997	1.724	13.208
	Moisture	0.546	0.562	0.990	0.536	0.532	0.988	0.574	0.989	0.576	6.678
	Ash	0.335	0.344	0.961	0.333	0.338	0.966	0.359	0.955	0.361	3.245
	Protein	3.315	3.409	0.950	3.338	3.431	0.936	3.513	0.944	3.525	2.886
	Carbohydrate	3.780	3.887	0.980	3.734	3.780	0.949	3.979	0.978	3.978	4.666
PCR	Oil	1.597	1.643	0.998	1.573	1.501	0.997	1.706	0.997	1.690	13.492
	Moisture	0.540	0.556	0.990	0.543	0.519	0.990	0.578	0.989	0.580	6.702
	Ash	0.375	0.385	0.951	0.379	0.387	0.942	0.400	0.944	0.401	3.037
	Protein	3.858	3.967	0.932	3.895	4.046	0.811	4.078	0.924	4.091	2.608
	Carbohydrate	4.280	4.401	0.974	4.373	4.633	0.948	4.550	0.971	4.554	4.175

**Table 2:** The nutrition of candlenut (cnd), peanut (pnt), sesame (ssm), sunflower (snf), sacha inchi residue (inc), and black bean (bkb).

Methods	Nutrition (%)	cnd	pnt	ssm	snf	inc	bkb	rdb

NIR Spectroscopy	Oil	60.956^∗^	39.733^∗^	56.288^∗^	44.390^∗^	20.941^∗^	4.274^∗^	0.237^∗^
		±1.279	±1.197	±1.791	±1.542	±2.349	±0.440	±0.084
	Moisture	4.329^∗^	7.146^∗^	3.550	3.086	10.485^∗^	8.927^∗^	14.388^∗^
		±0.419	±0.483	±0.402	±0.305	±0.874	±0.571	±0.526
	Ash	1.720^∗^	2.370^∗^	2.726	4.440	5.187^∗^	4.293	2.703
		±0.676	±0.124	±0.138	±0.067	±0.307	±0.059	±0.164
	Protein	20.521	15.586^∗^	21.543	28.549	47.865^∗^	31.398	18.952
		±2.273	±0.986	±3.341	±3.702	±5.142	±3.660	±1.768
	Carbohydrate	12.438	35.186^∗^	15.945	19.542^∗^	15.519	51.109^∗^	63.687^∗^
		±2.518	±1.550	±3.342	±3.879	±5.602	±3.744	±1.996

NMR Spectroscopy	Omega 3	23.310	nd	nd	nd	44.050	10.232	
	Omega 6	42.046	36.040	43.591	58.752	33.792	45.457	
	Omega 9	22.480	41.102	37.489	21.712	10.842	26.796	
	SFA	12.164	22.858	18.920	19.536	11.316	17.515	
	PUFA	65.356	36.040	43.591	58.752	77.842	55.688	

GC-FID	Methyl butyrate	nd	0.391	nd	nd	nd	nd	
	Methyl palmitate	6.341	17.529	9.481	12.051	4.152	10.328	
	Methyl stearate	3.022	1.761	5.205	9.206	2.944	3.885	
	Methyl oleate	22.887	23.455	38.422	38.467	8.070	25.455	
	Methyl linoleate	43.003	50.366	46.345	36.939	40.139	51.627	
	Methyl arachidate	nd	2.006	0.554	0.600	nd	nd	
	Methyl gamma linolenate	24.747	0.923	nd	1.102	44.301	8.704	
	Methyl behenate	nd	3.567	nd	1.632	0.395	nd	
	SFA	9.364	25.254	15.240	23.488	7.491	14.213	
	PUFA	67.750	51.289	46.345	38.040	84.440	60.331	

LC-MS/MS	Resveratrol	1.066	1.457	2.746	1.888^∗^	2.467	1.269	
		±0.067	±0.100	±0.155	±0.101	±0.134	±0.066	

HPLC-ELSD	Xylose	81.610	94.510^∗^	80.970	81.770	84.270	80.830	
		±1.260	±6.690	±0.132	±1.230	±2.920	±0.286	
	Fructose	95.100	462.270	83.090	80.140	967.780^∗^	79.100	
		±7.120	±22.870	±2.820	±1.520	±396.050	±0.268	
	Sorbitol	163.700	158.720	171.990^∗^	158.730	158.520	158.570	
		±4.290	±0.517	±1.530	±0.150	±0.336	±0.100	
	Galactose	223.000	533.750^∗^	210.260	209.760	226.530	209.630	
		±23.220	±26.600	±0.515	±0.235	±25.490	±0.145	
	Glucose	96.430	563.860	77.080	71.330	961.220	64.310	
		±34.650	±47.680	±1.860	±1.930	±421.290	±0.461	
	Sucrose	1429.500	116.660	1506.260	2416.240	2485.080	158.740	
		±88.280	±51.870	±432.570	±63.640	±192.310	±54.730	
	Lactose	132.780	136.860	132.500	179.480	141.220	131.680	
		±1.500	±2.750	±1.080	±42.610	±16.450	±0.015	
	Maltose	30.630	34.990	33.140	34.500	30.890	30.780	
		±0.043	±1.530	±3.720	±6.390	±0.185	±0.110	

^∗^significant at the 0.05 level; ±standard deviation; nd (not detected); red bean (rdb) and GC-FID were additional data; resveratrol in *μg/100* g *oil*; sugar in *mg/100 g flour.*

## Data Availability

The authors confirm that the data supporting this study's findings are available within the article.

## Abbreviations

RMSEC: Root mean square error of calibration
SEC: Standard error of calibration
R^2^C: Coefficient of determination in calibration
RMSECV: Root mean square error of cross-validation
SECV: Standard error of cross-validation
R^2^CV: Coefficient of determination in cross-validation
RMSEP: Root mean square error of prediction
SEP: Standard error of prediction
R^2^P: Coefficient of determination in prediction
RPD: Residual predictive deviation
PUFA: Polyunsaturated fatty acids
SFA: Saturated fatty acids
UFA: Unsaturated fatty acids
^1^H NMR: Proton nuclear magnetic resonance
NIR: Near-infrared
LC-MS/MS: Liquid chromatography with tandem mass spectrometry
TIC: Total ion chromatogram
MRM mode: Multiple reaction monitoring
HPLC-ELSD: High-performance liquid chromatography-evaporative light scattering detection
GC-FID: Gas chromatography-flame ionization detection.
